# RETRACTION: Effects of Cluster Nursing Interventions on the Prevention of Pressure Ulcers in Intensive Care Units Patients: A Meta‐Analysis

**DOI:** 10.1111/iwj.70456

**Published:** 2025-03-26

## Abstract

**RETRACTION:** ZhangA.‐G. , LiL. , LiY.‐L. , SunS.‐X. , WangM. , and TangX.‐L. , “Effects of Cluster Nursing Interventions on the Prevention of Pressure Ulcers in Intensive Care Units Patients: A Meta‐Analysis,” International Wound Journal 21, no. 3 (2024): e14776, 10.1111/iwj.14776.38494661 PMC10945037

The above article, published online on 17 March 2024, in Wiley Online Library (http://onlinelibrary.wiley.com/), has been retracted by agreement between the journal Editor in Chief, Professor Keith Harding; and John Wiley & Sons, Ltd. Following an investigation by the publisher, all parties have concluded that this article was accepted solely on the basis of a compromised peer review process. The editors have therefore decided to retract the article. The authors did not respond to our notice regarding the retraction.

**RETRACTION:** A.‐G. Zhang , L. Li , Y.‐L. Li , S.‐X. Sun , M. Wang , and X.‐L. Tang , “Effects of Cluster Nursing Interventions on the Prevention of Pressure Ulcers in Intensive Care Units Patients: A Meta‐Analysis,” International Wound Journal 21, no. 3 (2024): e14776, 10.1111/iwj.14776.38494661 PMC10945037

The above article, published online on 17 March 2024, in Wiley Online Library (http://onlinelibrary.wiley.com/), has been retracted by agreement between the journal Editor in Chief, Professor Keith Harding; and John Wiley & Sons, Ltd. Following an investigation by the publisher, all parties have concluded that this article was accepted solely on the basis of a compromised peer review process. The editors have therefore decided to retract the article. The authors did not respond to our notice regarding the retraction.

